# Liraglutide protects against glucolipotoxicity‐induced RIN‐m5F β‐cell apoptosis through restoration of PDX1 expression

**DOI:** 10.1111/jcmm.13967

**Published:** 2018-10-24

**Authors:** Edy Kornelius, Hsin‐Hua Li, Chiung‐Huei Peng, Yi‐Sun Yang, Wei‐Jen Chen, Yan‐Zin Chang, Yi‐Chiao Bai, Stanley Liu, Chien‐Ning Huang, Chih‐Li Lin

**Affiliations:** ^1^ Division of Endocrinology and Metabolism Department of Internal Medicine Chung Shan Medical University Hospital Taichung Taiwan; ^2^ Institute of Medicine Chung Shan Medical University Taichung Taiwan; ^3^ Division of Basic Medical Science Hungkuang University Taichung Taiwan; ^4^ Department of Biomedical Sciences Chung Shan Medical University Taichung Taiwan; ^5^ Department of Medical Research Chung Shan Medical University Hospital Taichung Taiwan

**Keywords:** glucolipotoxicity, liraglutide, Mst1, PDX1, β‐cell

## Abstract

Prolonged exposure to high levels of glucose and fatty acid (FFA) can induce tissue damage commonly referred to as glucolipotoxicity and is particularly harmful to pancreatic β‐cells. Glucolipotoxicity‐mediated β‐cell failure is a critical causal factor in the late stages of diabetes, which suggests that mechanisms that prevent or reverse β‐cell death may play a critical role in the treatment of the disease. Transcription factor PDX1 was recently reported to play a key role in maintaining β‐cell function and survival, and glucolipotoxicity can activate mammalian sterile 20‐like kinase 1 (Mst1), which, in turn, stimulates PDX1 degradation and causes dysfunction and apoptosis of β‐cells. Interestingly, previous research has demonstrated that increased glucagon‐like peptide‐1 (GLP‐1) signalling effectively protects β cells from glucolipotoxicity‐induced apoptosis. Unfortunately, few studies have examined the related mechanism in detail, especially the role in Mst1 and PDX1 regulation. In the present study, we investigate the toxic effect of high glucose and FFA levels on rat pancreatic RINm5F β‐cells and demonstrate that the GLP‐1 analogue liraglutide restores the expression of PDX1 by inactivating Mst1, thus ameliorating β‐cell impairments. In addition, liraglutide also upregulates mitophagy, which may help restore mitochondrial function and protect β‐cells from oxidative stress damage. Our study suggests that liraglutide may serve as a potential agent for developing new therapies to reduce glucolipotoxicity.

## 1 INTRODUCTION

Metabolic syndrome is a collection of symptoms that can cause disruption of the metabolism of carbohydrates, fats and proteins. Although the exact cause of metabolic syndrome is not fully understood, insulin resistance in obesity is recognized to be its main underlying cause.^1^, ^2^ Insulin resistance is a known direct cause of hyperglycaemia, and obesity is strongly linked to hyperlipidemia; both hyperglycaemia and hyperlipidemia lead to a higher risk of developing type 2 diabetes (T2D).^3^ Prolonged exposure to hyperglycaemia and hyperlipidemia can induce cell and tissue damage, also known as glucotoxicity and lipotoxicity respectively.^4^ Interestingly, hyperlipidemia is characterized by an increase in circulating free fatty acids (FFA), which is recognized as a major driving force for insulin resistance and results in constantly high blood sugar levels.^5^ Thus, hyperglycaemia seems to work synergistically with hyperlipidemia via a process referred to as glucolipotoxicity, which contributes to various cellular dysfunctions, including endoplasmic reticulum (ER) stress, oxidative stress, mitochondrial dysfunction and chronic low‐grade inflammation.^6^ Evidence has emerged that elevated serum FFA is implicated in stimulating cell dysfunction or cell death events, such as insulin resistance in skeletal muscle cells, fatty liver and steatosis in hepatocytes, and dysregulated insulin secretion and apoptosis in β‐cells.^7^ Although some studies demonstrate that lipotoxicity‐induced apoptosis is a specific effect of saturated FFA, unsaturated FFA is also toxic in an alternative pathway, which suggests that both saturated and unsaturated FFA markedly differ in their contributions to lipotoxicity.^8^ Therefore, the combination of both types of FFAs may best reflect the physiological or pathological situation under glucolipotoxicity.^9^

Research has indicated that β‐cells are particularly sensitive to glucolipotoxicity.^10^ In fact, pancreatic β‐cell dysfunction and death are two core features of the late stages of T2D.^11^ In diabetics, β‐cells secrete larger‐than‐normal amounts of insulin to overcome insulin resistance, causing overwork and overproduction of insulin for extended periods of time. Glucolipotoxicity also damages β‐cells, leading to little or no insulin production in a vicious cycle that ultimately promotes cell dysfunction and death.^12^ Thus, glucolipotoxicity‐mediated β‐cell loss is a critical causal factor of the late stages of diabetes. Although the exact mechanisms have not been delineated, studies have indicated that high glucose and FFA‐induced metabolic stress appears to regulate β‐cell identity and fate by perturbation of some specific transcription factors.^13^ In particular, pancreatic duodenal homeobox 1 (PDX1) has been reported to play a key role in maintaining β‐cell function and survival.^14^ Decreased expression of PDX1 can be found in β‐cells isolated from T2D patients, which suggests that PDX1 is important in islet compensation for glucolipotoxicity‐induced insulin resistance.^15^ Similarly, prolonged exposure of β‐cells to high glucose and FFA levels has been demonstrated to stimulate PDX1 nuclear exclusion and degradation, resulting in decreased insulin gene transcription and cell survival.^13^ On the contrary, recent studies reveal that administration of a glucagon‐like peptide 1 (GLP‐1) analogue, liraglutide, ameliorates the impairments to β‐cells by upregulation of PDX1 in high‐fat diet‐induced diabetic mice.^16^ Therefore, upregulation of PDX1 may prevent the progression of the glucolipotoxicity‐induced β‐cell dysfunction found in T2D. These previous findings highlight the importance of understanding how PDX1 is regulated.

Ardestani et al showed that mammalian sterile 20‐like kinase 1 (Mst1) can stimulate ubiquitin‐proteasome degradation of PDX1 and prohibit its function as a transcription factor in the nucleus.^17^ This result indicates that regulation of Mst1 and PDX1 may be involved in liraglutide‐mediated β‐cell protection. However, few studies have examined this mechanism in detail. Therefore, in the present study, we investigate the effect of high glucose and FFA treatment on rat pancreatic RINm5F β‐cells. Our results demonstrate that liraglutide restores the expression of PDX1 by inactivating Mst1, thereby ameliorating the impairments to RINm5F cells due to glucolipotoxicity. In addition, liraglutide also upregulates mitophagy to restore mitochondrial function and protect β‐cells from glucolipotoxicity. Our findings suggest that activation of GLP‐1 signalling by liraglutide may serve as an agent for the development of new therapeutic strategies against metabolic syndrome and T2D.

## 2 MATERIALS AND METHODS

### 2.1 Materials

We purchased reagents, such as palmitic acid (PA), oleic acid (OA), 3‐(4,5‐dimethylthiazol‐2‐yl)‐2,5‐diphenyltetrazolium bromide (MTT), 4′,6‐diamidino‐2‐phenylindole (DAPI), acridine orange (AO), dihydroethidium (DHE) and 2′,7′‐dichlorodihydrofluorescein diacetate (H_2_‐DCFDA), from Sigma‐Aldrich (München, Germany). Mst1 siRNA (#AM16708) was purchased from Thermo Fisher Scientific (Waltham, MA, USA). We purchased antibodies against caspase 3, poly(ADP‐ribose) polymerase (PARP), PDX1, Mst1, Lamin B1, p‐PERK, p‐eIF2α, AMPK, p‐AMPK, Nrf2 and PGC1α from Santa Cruz Biotechnology (Santa Cruz, CA, USA). Antibodies against LC3 and β‐actin were obtained from Novus Biologicals (Littleton, CO, USA), and antibodies against SOD1 and Sirt1 were purchased from GeneTex (Irvine, CA, USA). Liraglutide was purchased from Novo Nordisk (Copenhagen, Denmark). All chemicals were dissolved in phosphate buffer saline (PBS) solution and stored at −20°C until used in the experiments.

### 2.2 Cell culture, siRNA transfection and MTT assay

Rat RINm5F β‐cells were obtained from the American Type Culture Collection (Bethesda, MD, USA); maintained in RPMI‐1640 (Gibco, Carlsbad, CA, USA) supplemented with 10% fetal calf serum, 100 units/mL penicillin, 100 μg/mL streptomycin and 2 mmol L^−1^ l‐glutamine; and kept at 37°C in a humidified atmosphere of 5% CO_2_. For siRNA transfection, synthetic Mst1 siRNA was employed to knockdown target gene levels and protein expression by using Lipofectamine 2000 (Thermo Fisher Scientific) according to the manufacturer's instructions. For MTT viability tests, cells were seeded in 24‐well plates overnight and then treated under the indicated conditions. After treatment, MTT was added to the medium in accordance with the manufacturer's instructions. Only viable cells can metabolize MTT into a purple formazan product, the optical density of which was quantified by a Jasco V‐700 spectrophotometer (JASCO, Tokyo, Japan) at 550 nm. The average population number of control cells was set to 100% to enable comparison of the survival rates of other tested cells.

### 2.3 Western blot analysis

Cells were harvested and homogenized with protein extraction lysis buffer containing 50 mmol L^−1^ Tris‐HCl at pH 8.0, 5 mmol L^−1^ ethylenediaminetetraacetic acid, 150 mmol L^−1^ sodium chloride, 0.5% Nonidet P‐40, 0.5 mmol L^−1^ dithiothreitol, 1 mmol L^−1^ phenylmethylsulfonyl fluoride, 0.15 units/mL aprotinin, 5 μg/mL leupeptin, 1 μg/mL pepstatin and 1 mmol L^−1^ sodium fluoride. The solution was centrifuged at 12 000 × *g* for 30 minutes at 4°C to remove debris, and the supernatant cell lysate was used for immunoblotting analysis.

In order to isolate the nuclear and cytosolic fractions, cell extracts were made by using NE‐PER Nuclear and Cytoplasmic Extraction Kit (Thermo Fisher Scientific) according to the manufacturer's instructions. Equal amounts (50 μg) of total proteins from the cell lysate were resolved through SDS‐PAGE, transferred onto polyvinylidene difluoride membranes (Millipore, Bedford, MA, USA), and then probed with a primary antibody followed by another secondary antibody conjugated with horseradish peroxidase. Primary antibodies were used at a dilution of 1:1000 in 0.1% Tween‐20, and secondary antibodies were used at a dilution of 1:5000. Immunocomplexes were visualized using enhanced chemiluminescence kits (Millipore). The relative expression levels of proteins were densitometrically quantified using ImagePro Plus 6.0 software (Media Cybernetics, Silver Spring, MD, USA), further normalized on the basis of the expression level of the housekeeping protein β‐actin, and then compared with the normalized protein levels of control cells. The control protein level was set to 100% for comparison.

### 2.4 Assessment of nuclear morphology through DAPI staining

Changes in cell nuclear morphology characteristic of apoptosis were examined by fluorescence microscopy. Cells were fixed in 4% paraformaldehyde after 24 hours of treatment with the indicated compounds, permeabilized in ice‐cold methanol, incubated for 15 minutes with 1 ng/mL DAPI stain at room temperature, and then observed under a fluorescence microscope (DP80/BX53; Olympus, Tokyo, Japan). Apoptotic cells were quantified by counting five random fields per treatment.

### 2.5 mRNA expression analysis through reverse‐transcription quantitative PCR

Total mRNA was extracted using the RNeasy Kit (Qiagen, Germantown, MD, USA) and quantified spectrophotometrically. mRNA was reverse transcribed to cDNA by using TProfessional Thermocycler Biometra (Göttingen, Germany) under the following conditions: primer binding at 25°C for 10 minutes, reverse transcription at 37°C for 120 minutes and reverse transcriptase denaturation at 85°C for 5 minutes. mRNA was quantified through reverse‐transcription quantitative PCR (qPCR) with the ABI 7300 Sequence Detection System (Applied Biosystems, Foster City, CA, USA). Target genes were amplified by using Power SYBR Green PCR Master Mix (Applied Biosystems) in accordance with the manufacturer's instructions. Each cDNA sample was tested in triplicate. The following temperature parameters were used: initial denaturation at 95°C for 10 minutes; 40 cycles of denaturation at 95°C for 15 seconds; annealing at 60°C for 1 minute; and dissociation at 95°C for 15 seconds, 60°C for 15 seconds and 95°C for 15 seconds. The following primer pairs were used: forward 5′‐ACA CCT GTG CGG CTC ACA‐3′ and reverse 5′‐TCC CGG CGG GTC TTG‐3′ for insulin; and forward 5′‐TGG TAT CGT GGA AGG ACT CAT GAC‐3′ and reverse 5′‐ATG CCA GTG AGC TTC CCG TTC AGC‐3′ for GAPDH. The values of relative mRNA expression were obtained by using Sequence Detection Systems software (Sequence Detection Systems 1.2.3‐7300 Real‐Time PCR System; Applied Biosystems) and standardized by comparison with those obtained for the relative expression of GAPDH.

### 2.6 ELISA to determine insulin levels

Cells were seeded overnight in 6‐well plates and treated as indicated. Insulin levels in culture medium were quantified using an insulin rat ELISA kit (Invitrogen, Carlsbad, CA, USA) according to the manufacturer's instructions.

### 2.7 Analysis of mitochondrial transmembrane potential (Δψm)

Vital mitochondrial cationic dye JC‐1 was used to investigate mitochondrial function; this dye exhibits potential‐dependent accumulation in mitochondria. In normal cells, JC‐1 exists as a monomer and produces red fluorescence. During induction of Aβ cytotoxicity, the mitochondrial transmembrane potential collapses, and JC‐1 forms aggregates that produce red fluorescence. After treatment under the indicated conditions, cells were treated in fresh medium containing 1 μmol L^−1^ JC‐1 and incubated at 37°C for 30 minutes in an incubator. After discarding the staining medium and washing, cell imaging was performed using an inverted fluorescence microscope (DP72/CKX41; Olympus). Image Pro Plus 6.0 (Media Cybernetics, Rockville, MD, USA) software was used to measure the average fluorescence intensity of red and green fluorescence in each group, and results are presented as the ratio of average red/green fluorescence intensity. Five random, non‐adjacent fields in each group were used for statistical analysis.

### 2.8 Immunocytochemistry

After treatment, cells were fixed with 2% buffered paraformaldehyde, permeabilized in 0.25% Triton X‐100 (Sigma‐Aldrich) for 5 minutes at 4°C, and then incubated with anti‐αSyn primary antibody. Slides were incubated with an FITC‐labeled second antibody (Santa Cruz) in accordance with the origin of the primary antibody. Cells were stained with 1 μg/mL AO for 15 minutes and then washed with RPMI‐1640 medium. Thereafter, images were acquired by using a fluorescence microscope (DP80/BX53; Olympus) and cellSense V 1.9 digital imaging software.

### 2.9 Immunocytochemistry staining and AO staining

After treatment, cells were fixed with 2% buffered paraformaldehyde, permeabilized in 0.25% Triton X‐100 (Sigma‐Aldrich) for 5 minutes at 4°C and then incubated with anti‐PDX1 primary antibody. The slides were incubated with an FITC‐labeled second antibody (Santa Cruz) depending on the origin of the primary antibody. For AO staining, cells were stained with 1 μg/mL AO for 15 minutes and then washed with RPMI‐1640 medium. Images of the cells were acquired by using a fluorescence microscope (DP80/BX53; Olympus) and cellSense V 1.9 digital imaging software. The percentage of cells positive for red acidic vesicular organelles (AVOs) was calculated from five non‐adjacent images taken of each treatment.

### 2.10 Detection of superoxide thorough dihydroethidium staining

Intracellular superoxide levels were measured through DHE staining. DHE is a cell‐permeable fluorogenic probe that reacts with superoxide to form ethidium and emits red fluorescence. After treatment under the indicated conditions, the cells were treated in fresh medium containing 10 μmol L^−1^ DHE and incubated for 30 minutes in the dark at room temperature. After incubation, the staining medium was discarded, and the cells were washed twice with PBS. Cell imaging was then performed by using an inverted fluorescence microscope (DP72/CKX41; Olympus).

### 2.11 Measurement of reactive oxygen species by H_2_‐DCFDA

To evaluate intracellular reactive oxygen species (ROS) levels by using the cell‐permeant H_2_‐DCFDA method, cells were treated with 10 μmol L^−1^ H_2_‐DCFDA for 30 minutes. Intracellular oxidative burst was then measured using a NovoCyte flow cytometer (NovoCyte 2000; ACEA Biosciences, San Diego, CA, USA), and data analysis was performed using NovoExpress Software.

### 2.12 SA‐β‐galactosidase staining

SA‐β‐galactosidase catalyses the hydrolysis of X‐gal, which colours senescent cells blue. Cells were seeded in 6‐well plates overnight and then treated under the indicated conditions. After treatment, cells were rinsed with 1 mmol L^−1^ MgCl_2_ in PBS (pH 6.0) and then stained for SA‐β‐galactosidase using a senescence β‐galactosidase staining kit (Cell Signaling Technology, Danvers, MA, USA) according to the manufacturer's instructions. Finally, cells were washed twice with PBS, and images were taken using a microscope (BX53; Olympus) and cellSense V 1.9 digital imaging software. SA‐β‐galactosidase positive cells were quantified by counting five random fields per treatment.

### 2.13 Statistical analysis

All data are presented as means ± standard error of the mean. The statistical significance of differences between compared groups was determined through one‐way analysis of variance following Dunnett's posthoc test for multiple comparisons with SPSS Statistics version 22.0 software (SPSS Inc., Chicago, IL, USA), as well as the two‐tailed Student's *t* test. A probability value of <0.05 or <0.01 was specified to indicate statistical significance, and significance levels of *P < 0.05 or **P < 0.01 were set depending on individual experiments.

## 3 RESULTS

### 3.1 Exposure of RINm5F β‐cells to high glucose and FFA levels increases apoptosis

**Figure 1:**
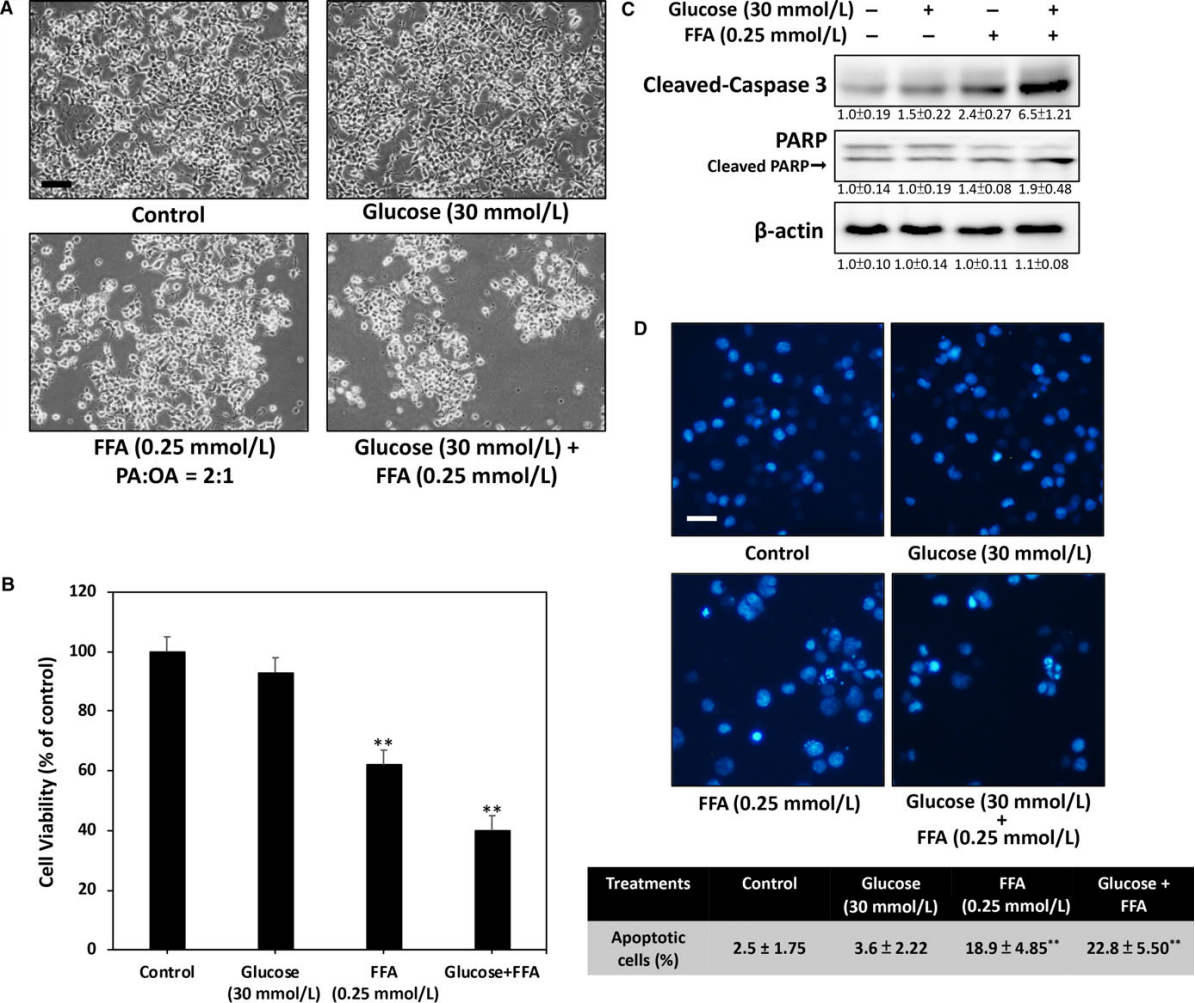
High glucose and high FFA induced apoptosis of rat RINm5f β‐cells. A, Phase‐contrast microscopic images of cells taken after 24 hours of treatment. Treatment of RINm5f cells with high glucose did not induce morphological changes and toxicity. However, high FFA or high glucose + FFA treatment markedly inhibited cell viability. B, MTT assay results indicated 38% cell death in the high FFA group and 60% cell death in the glucose + FFA group after 24 h of treatment relative to those in the control and high glucose groups. C, Results of Western blot analysis demonstrated that 24 h of treatment of glucose + FFA stimulates caspase 3 and PARP activation in RINm5f cells. D, Nuclear condensation and fragmentation were markedly increased in both high FFA‐ and high glucose + FFA‐treated cells compared with those in the control and high glucose‐treated cells after 24 h. Results were determined on the basis of condensed and fragmented nuclear morphology through DAPI fluorescence. All data were collected from at least three independent experiments, and values are presented as mean ± SEM. Significant differences were determined through multiple comparisons with Dunnett's posthoc test at *P < 0.05 and **P < 0.01 compared with the control groups. Scale bar = 20 μm

Elevated glucose and FFA levels in the circulation play a pivotal role in causing β‐cell damage. To fit the actual physiological composition of FFA, we used two common saturated and unsaturated dietary lipids, ie PA and OA, to establish a high‐FFA condition. Figure 1A shows the morphological change achieved by treating RINm5f β‐cells with high glucose, high FFA or a combination of the two for 24 hours. The results indicate that high glucose (30 mmol L^−1^) does not induce significant toxicity. By contrast, high FFA (0.25 mmol L^−1^, PA:OA = 2:1) markedly increased cell death, which was further enhanced in cells treated with the combination of high levels of glucose and FFA. The MTT assays showed 35% cell death in the high FFA group and 60% cell death in the glucose + FFA group (Figure 1B). These observations were confirmed by Western blot analysis, which showed that co‐treatment of glucose + FFA in RINm5f β‐cells for 24 hours increases the cleavage of caspase 3 and PARP, two typical apoptosis markers (Figure 1C). To determine the mode of cell death induced by high glucose + FFA, we used DAPI nuclear staining to investigate the occurrence of nuclear condensation and fragmentation. As shown in Figure 1D, nuclear fragmentations were significantly enhanced in the high FFA and high glucose + FFA groups compared with those in the control and high glucose groups. These results collectively demonstrate that co‐treatment of high glucose and high FFA promotes apoptotic RINm5f β‐cell death.

### 3.2 Treatment with high glucose and high FFA represses PDX1 expression and blocks insulin synthesis in RINm5F β‐cells

**Figure 2:**
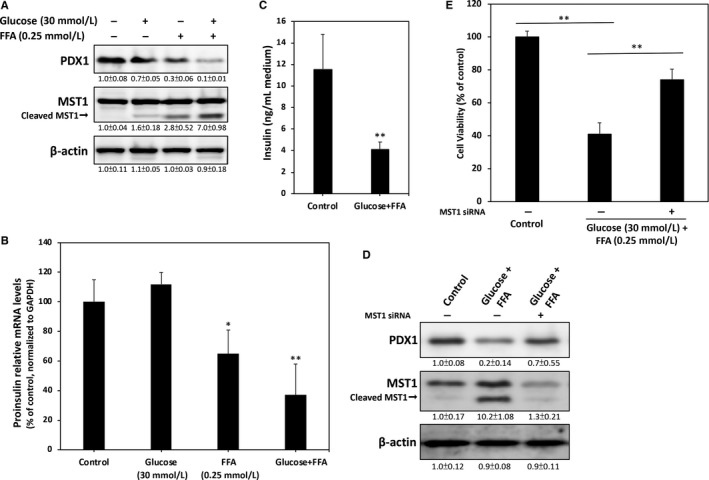
High glucose and FFA causes RINm5f β‐cell dysfunction through reduction of PDX1 expression. A, Immunoblotting revealed that the expression of PDX1 is clearly down‐regulated when cells are treated with high glucose and FFA for 24 hours. Conversely, the level of cleaved Mst1 markedly increased in the high glucose and FFA groups. B, Real‐time qPCR was used to measure proinsulin mRNA transcript levels. C, Treatment of cells with high glucose and high FFA for 24 h showed significantly decreased insulin secretion levels in the culture medium. D, High glucose and high FFA induced marked PDX1 reduction. However, knockdown of Mst1 by siRNA partially restored this inhibition. E, Cell viability was determined through MTT assay. Mst1 knockdown led to a significant restoration of cell survival. At least three independent experiments were performed, and values are presented as mean ± SEM. Significant differences were determined through multiple comparisons with Dunnett's posthoc test at *P < 0.05 and **P < 0.01 compared with the control groups

Previous studies have suggested that changes in PDX1 are linked to glucolipotoxicity‐induced β‐cell dysfunction and death. Activation of MST1 is also correlated with β‐cell failure by inhibition of PDX1. Therefore, we sought to determine whether co‐treatment of high glucose and high FFA alters the expression levels of PDX1 and Mst1 in RINm5f β‐cells. The immunoblotting results in Figure 2A reveal that high glucose alone does not induce inhibition of PDX1 expression levels. High FFA treatment, on the other hand, resulted in a marked decrease in PDX1 after 24 hours. This inhibition was further enhanced by co‐treatment with glucose, thereby indicating that exposure to high glucose and FFA for 24 hours strongly decreases PDX1 expression in RINm5f β‐cells. These results are in accordance with our findings on the expression of cleaved Mst1, which was markedly increased in the high glucose + FFA group. Given that Mst1 cleavage produces increased enzyme activity contributing to MST1‐induced PDX1 degradation, the results imply that the mechanism of MST1‐induced downregulation of PDX1 may be involved in glucolipotoxicity‐associated β‐cell failure.

To further elucidate whether high glucose + FFA treatment interferes with RINm5f β‐cell function during insulin synthesis and secretion, we performed relative expression qPCR assays to measure the levels of proinsulin mRNA transcripts. As shown in Figure 2B, no significant changes were found in the expression of proinsulin mRNA in the high‐glucose group compared with that in the control group. However, high FFA significantly suppressed mRNA levels of proinsulin, and this inhibition was enhanced by co‐treatment with high FFA. The amount of insulin in the medium was determined by ELISA, and results revealed that high glucose + FFA treatment strongly suppresses insulin secretion (Figure 2C).

To determine whether the observed glucolipotoxicity‐mediated β‐cell dysfunction is due to Mst1, we conducted knockdown experiments using Mst1 siRNA. As expected, our results showed a significant reduction in the expression levels of both non‐cleaved and cleaved Mst1 after transfection of anti‐Mst1 siRNA (Figure 2D). Results from cell viability assays also demonstrated that knockdown of Mst1 effectively alleviates high glucose + FFA‐induced cytotoxicity (Figure 2E). Taken together, the results of these experiments indicate that glucolipotoxicity may cause RINm5f β‐cell dysfunction through Msta1‐mediated reduction of PDX1.

### 3.3 Liraglutide protects against glucolipotoxicity‐induced RINm5f β‐cell death by restoring PDX1 nuclear levels

**Figure 3:**
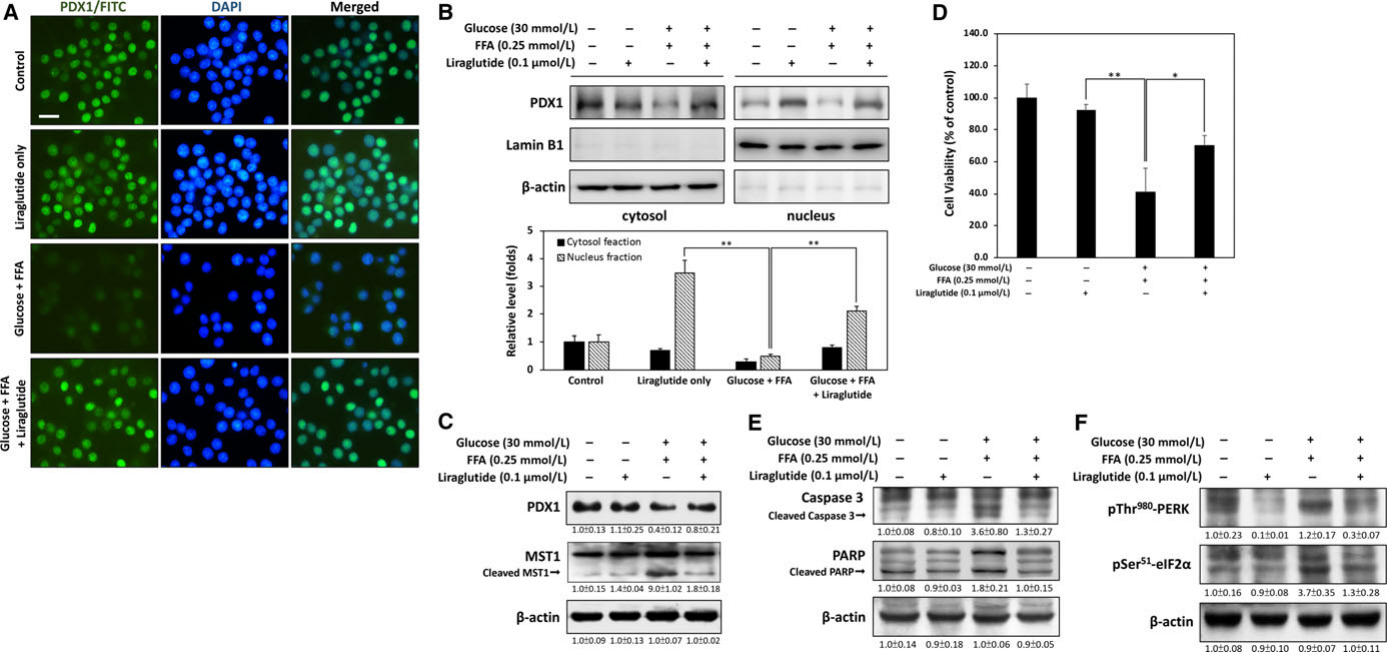
Liraglutide alleviates glucolipotoxicity‐induced cell death by restoring PDX1 protein levels. A, Immunofluorescence staining revealed that nuclear PDX1 protein levels were markedly reduced by treatment with high glucose and FFA for 24 h. Co‐treatment with liraglutide (0.1 μmol L^−1^) effectively restored the amount of nuclear PDX1. B, Western blot analysis of PDX1 from nuclear and cytosolic fractions from RINm5f β‐cell extracts. Results showed that nuclear fraction of PDX1 is significantly restored by liraglutide. C, Western blot analysis showed up‐regulation of PDX1 and downregulation of cleaved Mst1 by liraglutide after 24 h of treatment with high glucose and FFA. D, Cell viability was determined through MTT assay. Liraglutide effectively alleviated glucolipotoxicity‐induced β‐cell death. E, Western blot analysis demonstrated that liraglutide inhibits caspase 3 and PARP activation during glucolipotoxicity. F, Immunoblotting revealed that phosphorylation of p‐Thr^980^ PERK and p‐Ser^51^ eIF2α are downregulated when cells are treated with 0.1 μmol L^−1^ liraglutide for 24 h. All data were collected from at least three independent experiments, and values are presented as mean ± SEM. Significant differences were determined through multiple comparisons with Dunnett's posthoc test at *P < 0.05 and **P < 0.01 compared with the control groups

Previous studies have suggested that activation of GLP‐1 signalling can protect and improve pancreatic β‐cell function against glucotoxicity and lipotoxicity.^18^, ^19^ Given that GLP‐1 is commonly used in the treatment of diabetic hyperglycaemia, we investigated whether liraglutide, a GLP‐1 analogue approved and widely used in the treatment of T2D, protects against high glucose and high FFA‐induced glucolipotoxicity in RINm5f β‐cells. The results of immunofluorescence staining in Figure 3A demonstrate that nuclear PDX1 protein levels are markedly reduced by co‐treatment of high glucose and FFA for 24 hours. Treatment with liraglutide (0.1 μmol L^−1^) effectively restored nuclear PDX1 levels, which means the Mst1‐suppressed PDX1 pathway may be blocked by liraglutide. Indeed, cytosol and nuclear fractions of western blotting confirmed that the nuclear fraction of endogenous PDX1 is significantly restored by liraglutide (Figure 3B). Furthermore, the results in Figure 3C show that liraglutide markedly up‐regulates PDX1 protein levels in high glucose and high FFA‐treated cells. Moreover, cleaved and activated Mst1 protein was reduced by liraglutide. This result was also confirmed through MTT assays, which showed that liraglutide effectively alleviates high glucose and high FFA‐induced cytotoxicity (Figure 3D). Liraglutide further reduced the cleavage of caspase 3 and PARP (Figure 3E), thus indicating that it can protect against glucolipotoxicity‐induced β‐cell apoptosis by restoring Mst1‐suppressed PDX1 expression levels.

ER stress‐induced apoptosis plays a key role in PDX1‐deficient β‐cell during glucolipotoxicity.^20^, ^21^To determine whether liraglutide protects cells from ER stress‐induced cell death events, we measured two ER stress downstream typical markers, including PKR‐like endoplasmic reticulum kinase (PERK) and eukaryotic translation initiation factor 2α (eIF2α), which activates a signalling network called the unfolded protein response to trigger C/EBP homologous protein‐mediated apoptosis.^22^ As shown in Figure 3F, treatment with high glucose and high FFA for 24 hours significantly increased the phosphorylation of PERK on threonine 980 and eIF2α on serine 51. However, liraglutide inhibited levels of p‐Thr^980^ PERK and p‐Ser^51^ eIF2α in high glucose and high FFA co‐treated cells, which means glucolipotoxicity‐induced ER stress is returned by addition of liraglutide.

### 3.4 Liraglutide alleviates glucolipotoxicity‐induced oxidative stress and cellular senescence

Previous studies have suggested that oxidative stress occurs in β‐cells as a consequence of glucolipotoxicity.^23^ Therefore, we investigated whether liraglutide protects cells from glucolipotoxicity‐induced oxidative stress damage by using DHE probe, an indicator of superoxide. As shown in Figure 3A, treatment with high glucose and high FFA for 24 hours caused a marked increase of superoxide accumulation. However, this effect was counteracted by liraglutide treatment, thereby indicating that oxidative stress may be decreased.

**Figure 4:**
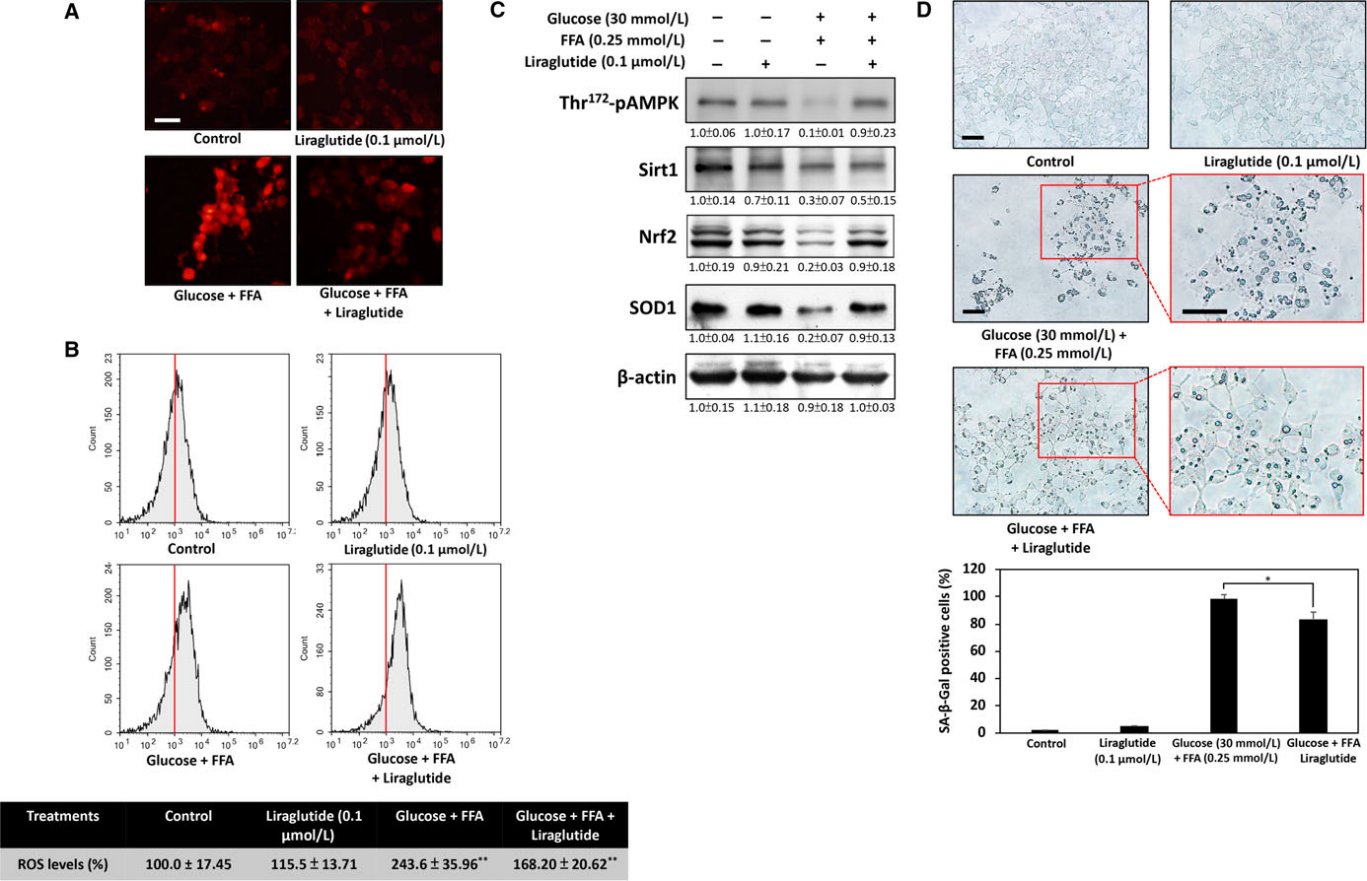
Liraglutide protects β‐cells against glucolipotoxicity‐induced oxidative stress and cellular senescence. A, Dihydroethidium staining results viewed under a fluorescence microscope show that liraglutide reduces high glucose and high FFA‐induced intracellular superoxide accumulation. B, Intracellular oxidative burst was determined by 2′,7′‐dichlorodihydrofluorescein diacetate using flow cytometric analysis. Liraglutide effectively reduced ROS levels in high glucose + FFA‐treated cells. C, Levels of some antioxidant signalling‐related proteins, including p‐AMPK/AMPK, Sirt1, Nrf2 and SOD1, as analysed through Western blot. D, Representative results of cytochemical detection of SA‐β‐galactosidase. SA‐β‐galactosidase positive cells are stained blue‐green, and staining intensity can be scored under a bright‐field microscope. All data were collected from at least three independent experiments, and values are presented as mean ± SEM. Significant differences were determined through multiple comparisons with Dunnett's posthoc test at *P < 0.05 and **P < 0.01 compared with the control groups. Scale bar = 20 μm

To measure oxidative stress levels accurately, we used cell‐permeant H_2_‐DCFDA as an indicator of ROS in cells. Upon flow cytometric analysis of DCFDA staining, significant up‐regulation of intracellular ROS accumulation occurred in response to treatment with high glucose and high FFA for 24 hours, and liraglutide effectively reduced ROS levels (Figure 3B). The cellular redox state is regulated by the sirtuin 1 (Sirt1)/nuclear factor erythroid 2‐related factor 2 (Nrf2) pathway to maintain the expression levels of endogenous antioxidant enzymes such as SOD1.^24^ AMP‐activated protein kinase (AMPK) mediation of Sirt1 activation has been proposed to stimulate this antioxidant defense response.^25^ As expected, our Western blot analysis results demonstrated that liraglutide treatment restores Sirt1 and Nrf2 levels and increases downstream superoxide dismutase 1 (SOD1), a cytosol ROS detoxifying enzyme, in high glucose + FFA‐treated cells (Figure 4C).

Some evidence suggests that glucolipotoxicity‐induced oxidative stress mediates cellular senescence, which contributes to β‐cell dysfunction.^26^ To investigate whether liraglutide can attenuate high glucose and high FFA‐induced senescence, we performed cytochemical SA‐β‐gal staining, a commonly used biomarker of senescent cells in culture. The results revealed a significant increase in the percentage of SA‐β‐galactosidase positive cells in high glucose and high FFA‐treated cells. Liraglutide attenuated these effects caused by glucolipotoxicity, thus suggesting that decreased ROS content by liraglutide may be responsible for protection against glucolipotoxicity‐induced cellular senescence.

### 3.5 Liraglutide restores glucolipotoxicity‐impaired mitophagy and mitochondrial membrane potential

**Figure 5:**
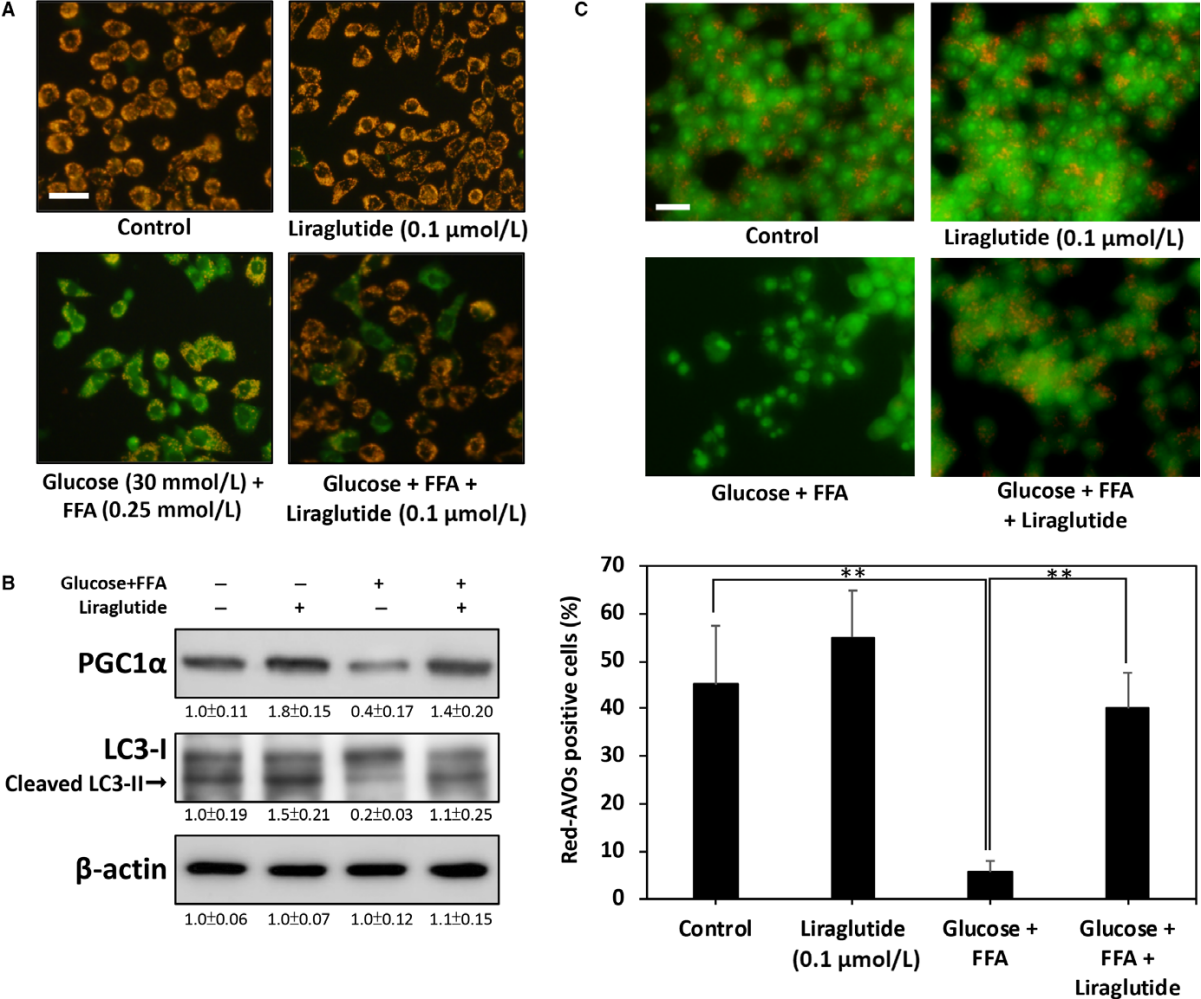
Liraglutide restores glucolipotoxicity‐impaired mitophagy and mitochondrial membrane potential. A, JC‐1 immunofluorescent staining. Green fluorescence represents the dissipation of mitochondrial membrane potential in high glucose + FFA‐treated RINm5f β‐cells after 24 h. Red fluorescence indicates that co‐treatment with liraglutide effectively preserves the mitochondrial membrane potential. B, Levels of PGC1α and LC3‐I/II protein in high glucose + FFA‐treated RINm5f β‐cells. Liraglutide markedly restored LC3‐II levels, thus suggesting that impaired mitophagy may be recovered. C, Representative images of AVO‐positive cells treated through AO staining. The percentage of red AVO‐positive cells was calculated from five images of each treatment. All data were collected from at least three independent experiments, and values are presented as mean ± SEM. The significance of differences was determined through multiple comparisons with Dunnett's posthoc test at *P < 0.05 and **P < 0.01 compared with the control groups. Scale bar = 20 μm

Glucolipotoxicity‐induced ROS accumulation and oxidative damage is caused by dysfunctional mitochondria.^27^ Thus, the role of liraglutide in preventing glucolipotoxicity‐mediated impairment of mitochondrial membrane potential was also investigated in this work. As shown in Figure 5A, exposure of cells to high glucose + FFA resulted in an increase in green fluorescence, indicating a great loss of mitochondrial membrane potential. However, co‐treatment with liraglutide reduced the effects of high glucose + FFA on mitochondrial membrane potential significantly, thus suggesting that liraglutide preserves mitochondrial function against glucolipotoxicity.

Proliferator‐activated receptor gamma coactivator 1 α (PGC1α) has been reported to be a positive regulator of mitochondrial function and renewal.^28^ Therefore, we investigated whether glucolipotoxicity down‐regulates PGC1α expression. As shown Figure 5B, high glucose + FFA caused a marked decrease in the expression of PGC1α at 24 hours post‐treatment, and this inhibition was effectively restored by co‐treatment of liraglutide.

Activation of PGC1α a and AMPK has been shown to enhance mitochondrial function and biogenesis by facilitating mitophagy.^29^ We therefore investigated whether inhibition of mitophagy by high glucose + FFA results in impairment of mitochondrial function. The results presented in Figure 5B show that treatment with high glucose + FFA for 24 hours causes markedly down‐regulated LC3‐II, a positive regulator in type 1 and type 2 mitophagy stimulation.^30^ Conversely, liraglutide co‐treatment significantly restored this down‐regulation, thus suggesting that impaired mitophagy may be recovered by liraglutide. The AO staining results also provided further evidence that high glucose + FFA significantly decreases the number of AVOs, a marker of autophagy/mitophagy. However, treatment with liraglutide significantly increased the percentage of cells containing AVOs from 5.6% to 40.0% (Figure 5C). Collectively, these results indicate that up‐regulation of mitophagy by liraglutide may help restore mitochondrial function and protect β‐cells from glucolipotoxicity.

## 4 DISCUSSION

Glucolipotoxicity, which refers to irreversible damage of chronically elevated levels of circulating glucose and fatty acid, plays an important role in the development of diabetes‐associated complications, particularly in inducing pancreatic β‐cell dysfunction and death.^31^ Many studies have demonstrated that long‐term exposure of β‐cells to high glucose and high FFA induces dysregulated insulin synthesis and secretion, eventually resulting in apoptosis. Thus, identification of the underlying mechanisms by which glucolipotoxicity contributes to β‐cell dysfunction and death is critical to develop novel therapeutic strategies aimed at delaying or preventing β‐cell exhaustion. Although the exact mechanism involved in β‐cell glucolipotoxicity has yet to be elucidated, several hypotheses have been proposed, including mitochondrial dysfunction, oxidative stress, ER stress and islet inflammation.^23^ However, the specific apoptotic signalling pathways involved in these mechanisms have not been comprehensively studied.

Ardestani et al recently demonstrated that Mst1 is a key regulator of β‐cell apoptosis and dysfunction in diabetes^17^; the group found that activated Mst1 directly phosphorylates the β‐cell transcription factor PDX1, resulting in ubiquitination and degradation of PDX1 leading to impairment of β‐cells. These observations confirm that inhibition of Mst1 is a potent target for β‐cell rescue. Interestingly, there are some reports showing that overexpression of PDX1 may stimulate β‐cell neogenesis. For example, it has been demonstrated that the up‐regulation of PDX1 is involved in β‐cell reprogramming and neogenesis in adult mice.^32^ Similarly, transgenic overexpression of PDX1 in STZ‐treated mice can generate new insulin‐producing islet cells.^33^ All these supported that the up‐regulation of PDX1 may act as a key factor for preservation of β‐cell in diabetes. In parallel with these findings, Sasaki et al reported that the activation of GLP‐1 signalling can also promote β‐cell neogenesis *in vitro*, suggesting that the use of liraglutide may be a useful strategy for preserving or restoring β‐cell function through up‐regulation of PDX1 expression. However, the details of which are still largely unknown, and further research is required to determine the effect of Pdx1 overexpression under glucolipotoxicity.

Our present results suggest that treatment with the GLP‐1 analogue liraglutide significantly restores insulin synthesis and enhances cell viability in high glucose‐ and high FFA‐treated β‐cells. Furthermore, liraglutide up‐regulates the AMPK and Sirt1 pathways, which improve mitochondrial dysfunction and, thus, contribute to reductions in oxidative stress and attenuation of glucolipotoxicity. These findings indicate that restoration of PDX1 by inhibition of Mst1 cleavage may play a protective role against glucolipotoxicity‐induced β‐cell damage. Interestingly, liraglutide has been demonstrated to promote pancreatic β‐cell survival through inhibition of caspase‐3 activation.^34^ Mst1 alone sufficiently induces caspase activation, and activation of the caspase machinery (particularly caspase‐3) may be essential to the cleavage of Mst1. Cleaved and active Mst1 upregulate downstream apoptotic signals in β‐cells, forming a positive feedback loop that amplifies caspase‐3 activation and boosts the cleavage of the kinase.^35^ Therefore, inhibition of caspase 3 by liraglutide may silence Mst1‐dependent PDX1 suppression and protecting β‐cells against glucolipotoxicity. Although our results can be partially rationalized as explained above, the precise mechanism of liraglutide‐mediated Mst1 inhibition requires further elucidation.

Mitophagy is the selective degradation of mitochondria by autophagy and has been the subject of intense research in biological processes ranging from cellular metabolism to senescence. Increased mitophagy is thought to accelerate mitochondrial turnover, which, in turn, appears to maintain mitochondrial function and quality.^36^ In diabetes, dysfunctional mitochondria have been linked to increased levels of ROS accumulation, which ultimately underlies β‐cell failure and apoptosis.^37^ Moreover, mitochondrial dysfunction and oxidative stress are largely involved in cellular senescence, thus suggesting that protecting and improving mitochondrial function may be a useful strategy for treating some oxidative stress‐associated diseases, including neurodegeneration, metabolic syndrome and diabetes. In our results, we demonstrated that, under glucolipotoxicity, liraglutide up‐regulates the AMPK/LC3 autophagy pathway, which drives the turnover of dysfunctional mitochondria in β‐cells. Interestingly, previous studies indicate that binding of GLP‐1 to its β‐cell receptor elevates PGC1α expression, which means activation of GLP‐1 signalling may improve β‐cell functions via enhanced mitochondrial performance.^38^ Our findings revealed that co‐treatment of liraglutide effectively restore glucolipotoxicity‐induced PGC1α downregulation. Recent evidence also suggests the diminished expression of PGC1α in humans with insulin resistance and diabetes.^39^ As PGC1α is the master regulator of mitochondrial turnover, restoration of mitophagy by liraglutide can prevent glucolipotoxicity toxicity.^40^ In the present study, we provide evidence of the ability of liraglutide to inhibit high glucose and high FFA‐induced β‐cell toxicity. This protective effect appears to be associated with Mst1 and PDX1‐related regulation and mitophagy activation. To the best of our knowledge, our group is the first to demonstrate the molecular mechanism of liraglutide against glucolipotoxicity‐induced β‐cell damage by the Mst1 and PDX1 pathway. Our results provide new insights into the potential use of incretin‐based agents, such as liraglutide, in the preservation of β‐cell function in metabolic syndrome and diabetes.

## DISCLOSURE

No actual or potential conflict of interest.
